# Prevalence and associated risk factors of current hepatitis C infection among U.S. general population and injection drug users aged 20–59 years: NHANES 2009–2018

**DOI:** 10.1371/journal.pone.0309345

**Published:** 2024-08-26

**Authors:** Harun Mazumder, Md Faruk Hossain, Pratibha Shrestha, Sultan Mahmud, Maidul Husain, Rebeka Ahmed

**Affiliations:** 1 Institute of Statistical Research and Training, University of Dhaka, Dhaka, Bangladesh; 2 School of Public Health, Louisiana State University Health Sciences Center, New Orleans, LA, United States of America; 3 School of Medicine, Washington University, St. Louis, MO, United States of America; 4 Maternal and Child Health Division, International Centre for Diarrhoeal Disease Research, Bangladesh (icddr,b), Dhaka, Bangladesh; 5 Bangabandhu Sheikh Mujibur Rahman Science and Technology University, Gopalganj, Bangladesh; 6 Department of Zoology, National University, Gazipur, Bangladesh; Centers for Disease Control and Prevention, UNITED STATES OF AMERICA

## Abstract

**Introduction:**

The people who inject drugs (PWID) are attributed to high-risk groups for transmission of the Hepatitis C virus (HCV). This study assessed the prevalence and associated factors of current HCV infection (CHI) among U.S. general population and PWID of ages between 20 and 59 years old.

**Methods:**

This study utilized cross-sectional data from the 2009–2018 National Health and Nutrition Examination Survey, conducting separate analyses for the U.S. general population, including PWID and non-PWID, as well as specific analyses focusing solely on PWID. The analytical methods included the estimation of CHI prevalence, Rao-Scott chi-square test to compare CHI-positive and CHI-negative groups, and univariate and multivariable logistic regressions models to evaluate the associated risk factors of CHI.

**Results:**

The prevalence of CHI among general population and PWID were 1% and 19%, respectively. Compared to non-PWID, the odds of CHI were significantly higher among PWID (OR = 32.6, 95% CI = 17.7–60.3) in general population. Among PWID, male vs. female (OR = 2.6, 95% CI = 1.1–5.9), adults aged 40–59 vs. 20–39 years old (OR = 2.9, 95% CI = 1.2–7.3), Non-Hispanic Black vs. White (OR = 4.6, 95% CI = 1.5–13.6), with high school diploma or less educational attainment vs. above college degree (OR = 3.5, 95% CI = 1.4–9.2) showed higher odds of having CHI.

**Conclusion:**

The prevalence of CHI was found to be higher among PWID especially those who were male, aged 40–59 years old, Non-Hispanic Black, and had lower educational attainment. Targeted intervention such as screening and awareness program among PWID population is recommended to reduce the burden of new HCV infections in the U.S.

## Introduction

Infection with the hepatitis C virus (HCV) is a major concern in the U.S. and globally which contributes to significant mortality and morbidity [1]. After contracting HCV acutely, approximately 50%-80% of people develop chronic hepatitis C, which has serious consequences such as liver fibrosis, cirrhosis, and even hepatocellular carcinoma (primary liver cancer) [2]. According to a World Health Organization (WHO) report published in June 2022, approximately 58 million people worldwide have HCV, with approximately 1.5 million new infections occurring each year [3]. In 2019, approximately 290,000 people died from chronic HCV-related complications, the majority of whom had hepatic cirrhosis or hepatocellular carcinoma [3].

During 2013–2016, approximately 3.7 million noninstitutionalized U.S. adults were reported to be HCV antibody positive (indicating current or past infection) and 2.4 million were HCV-RNA positive indicating current infection [4]. Furthermore, HCV-related deaths in the United States during that time were higher and outnumbered deaths from 60 other nationally notifiable infectious diseases in 2013 [5,6]. According to the Centers for Disease Control and Prevention (CDC), the incidence rate and death rate of HCV increased by 15% and 4%, respectively, in the United States alone in 2020 compared to 2019 [7]. The treatment for acute HCV infection upon diagnosis may reduce the burden of chronic HCV-related liver diseases substantially, particularly in high-risk groups. The current HCV infection (CHI) rate has been reported to be on the rise in the U.S. despite HCV being simply diagnosable and curable [8].

Because HCV is a blood-borne virus, it can be spread by sharing needles, syringes, or other drug-injection equipment. Several studies have found higher rates of HCV prevalence in specific populations, such as people who inject drugs (PWID) [9]. Globally, HCV prevalence has been estimated at over 50% among PWID [10]. Long-term injection drug use (IDU) is a major concern in the U.S., accounting for approximately 60% of new HCV infections [11] which ranges from 70% to 90% [12,13], while uninfected PWID can acquire HCV at rates ranging from 10% to 40% per year [14,15]. Lack of interventions, inadequate care, and non-sterile shared injections all contribute to the spread of blood-borne diseases such as HCV [10,13,16]. Furthermore, HCV-positive individuals with reported IDU history have a poor prognosis compared to non-PWID despite the availability of highly effective antiviral therapy. The ongoing IDU as well as the psychiatric and other medical disorders leads to poor treatment compliance and the possibility of reinfection, eventually making this group a poor candidate for HCV treatment [17].

While previous studies have focused on estimating the prevalence of HCV infection in the U.S. population [4,18], there is limited information available regarding the recent prevalence and associated risk factors of HCV infections, especially among PWID in the U.S. The lack of current literature may obscure the potential hazards linked to the transmission of HCV within this community. An evaluation of the current prevalence and associated sociodemographic and clinically relevant factors of HCV infection is necessary to inform prevention and care efforts. This study aims to determine the prevalence of CHI status among the general population, including both PWID and non-PWID, and separately among PWID in the U.S. based on the most recent nationally representative data. Another important objective of this study is to evaluate the potential risk factors associated with CHI in the same populations. Knowing the prevalence and risk factors of CHI will be crucial to policymakers in public health planning and policy decisions pertaining to HCV prevention and control in both the general population and PWID.

## Methods

### Study population

**Fig 1:**
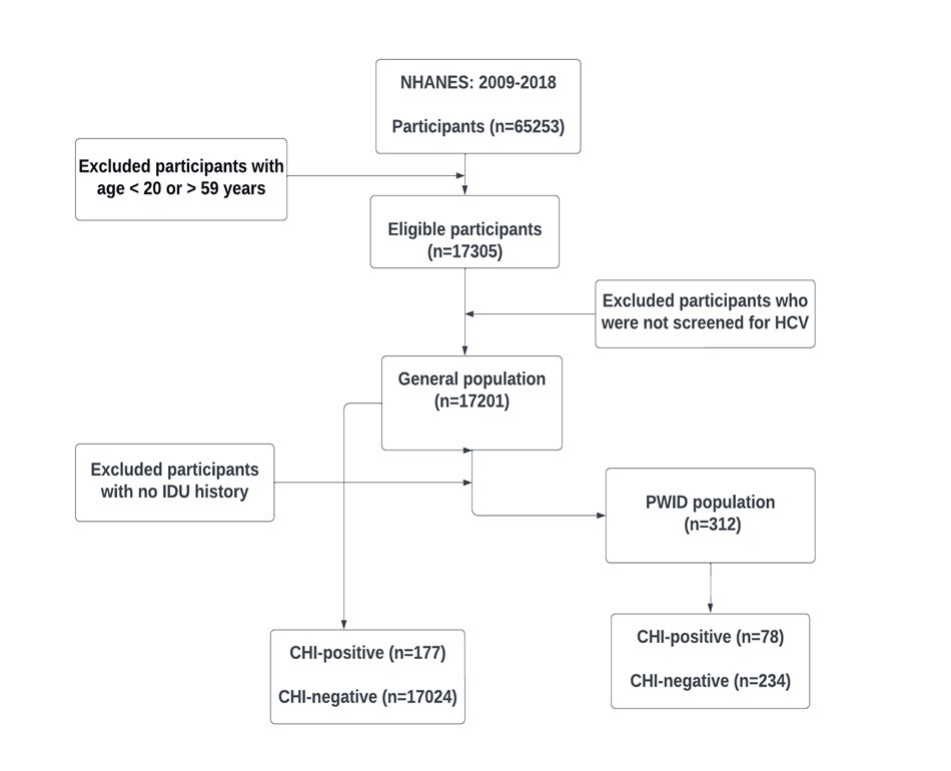
Flow diagram of analytical sample selection for general population and PWID.

Data were collected from National Health and Nutrition Examination Survey (NHANES) 2009–2018. NHANES uses the complex survey design to collect a representative sample of the U.S. population. More information on NHANES is provided elsewhere [19]. This study was not reviewed by the Institutional Review Board as the data analyzed were de-identified and publicly available. However, the National Center for Health Statistics (NCHS) Ethics Review Board did review and approve the NHANES protocol [20]. This study focused on the noninstitutionalized U.S. general population and PWID. It should be noted that the general population and PWID are not mutually exclusive. The general population included participants with or without a history of IDU, which allowed the evaluation risk attributes in PWID relative to non-PWID. Adults of ages 20–59 years old were included in the study since NHANES provides public access to IDU information only for this age group. Individuals lacking information on HCV screening were considered missing data and subsequently excluded from the study. The analytical sample size was 17201 for the general population, including 312 PWID participants (Fig 1). The 312 PWID participants were further analyzed in detail separately, without direct comparison to the general population. PWID was defined as those who responded “yes” to the question “Have you ever, even once, used a needle to inject a drug not prescribed by a doctor?”. Additional analyses defined PWID based on the response to the question “How long ago has it been since you last used a needle to inject a drug not prescribed by a doctor?” (PWID if within 12 months, non-PWID otherwise).

### Study variables

The response variable was the current HCV infection status (CHI): positive or negative. Participants diagnosed as positive in HCV RNA-test were identified as CHI “positive”. Predictors included: age (20–39, 40–59), gender (male, female), race (Hispanic, Non-Hispanic white, Non-Hispanic black, and others), education (high school graduate or less, some college degree, some college or above), poverty income ratio (<1: poor, 1–1.99: near poor, ≥2: not poor), insurance status (yes, no), history of blood transfusion (yes, no), history of IDU (yes, no), and hepatitis B virus (HBV) status (never infected, current/past infection, vaccine-induced immunity), number of sexual partners in last 12 months (0, 1, ≥2), HIV infection status (yes, no), and diabetes status (yes, no). The HBV status was created using the information on test results for HBV core antibody, surface antibody, and surface antigen using standard serological interpretation defined by CDC [21]. Participants with indeterminate core antibody tests were excluded due to the limited sample size.

### Laboratory testing

NHANES typically utilizes serological assays or kits, including methods such as Enzyme Immunoassay and chemiluminescence immunoassay, for HBV and HCV testing. While these tests are often automated to ensure precise and efficient laboratory analysis, there may be instances where manual methods were employed. The specific guidelines, serological kits, platforms, and automation status for HBV and HCV testing in NHANES may vary across survey cycles. Detailed information on laboratory methods, including HBV and HCV testing protocols, is accessible through the CDC NHANES website (https://wwwn.cdc.gov/nchs/nhanes/default.aspx) in the “Laboratory Methods” section under “Contents in Detail” for each NHANES cycle (e.g., NHANES 2015–16).

### Statistical analysis

All analyses in this study were adjusted by appropriate NHANES sampling weights (“WTMEC2yr”) to ensure nationally representative estimates [22]. The analyses were conducted in statistical software R using “*survey”* package [23]. The weighted percentages and corresponding 95% confidence intervals for risk factor characteristics by CHI status (positive, negative) were calculated. The Rao-Scott chi-square tests were conducted to evaluate differences in these characteristics between CHI status for categorical predictors, and the t-test for a continuous predictor. The prevalence of CHI positive by risk factor characteristics and corresponding 95% confidence intervals were calculated. To determine the risk factors of CHI, we performed univariate and multivariable logistic regression models. The above analyses were conducted for both the general population and PWID. A *P*-value less than 0.05 was considered statistically significant. The HBV status was excluded from the logistic regression analysis as the primary focus of risk factor assessment was not on the co-infection status.

## Results

### Characteristics of the study population

Table 1 displays the background characteristics of the study participants. Among 17,201 individuals from the general population, 177 tested positive for CHI, while 17,024 tested negative. Among the 312 PWID, 78 were CHI positive, and 234 were CHI negative. Among the CHI-positive individuals from the general population, 51% were female, and 50% belonged to the age group 20–39 years. The majority of CHI-positive individuals from the general population were Non-Hispanic White (61.7%). Additionally, 63.8% were not considered poor (Poverty Income Ratio ≥ 2), 78.7% had health insurance, 93.4% had not undergone blood transfusion, 68.3% had never been infected with Hepatitis B, 69% had one sexual partner in the past 12 months, 99.6% did not have HIV infection, and 93.8% did not have diabetes. Similarly, a large proportion of CHI-positive PWID were male (62.6%) and Non-Hispanic White (82.7%). Additionally, 48.1% had completed high school or lower education, 48.3% were not considered poor (Poverty Income Ratio ≥ 2), 69.2% had health insurance, 92.1% had not undergone blood transfusion, 68% had never been infected with Hepatitis B, 56% had one sexual partner in the past 12 months, and 93.8% were non-diabetic.

**Table 1:** Characteristics of study participants by CHI status in the general population and PWID.

	General Population				People who inject drugs			
		CHI (Positive)	CHI (Negative)			CHI (Positive)	CHI (Negative)	
**Characteristics**	**N** ^**a**^	**Wt% (95% CI)** ^**b**^	**Wt% (95% CI)** ^**b**^	**P-value** ^**c**^	**N** ^**a**^	**Wt% (95% CI)** ^**b**^	**Wt% (95% CI)** ^**b**^	**P-value** ^**c**^
Projected population		1524377 (N^a^ = 177)	156681459 (N^a^ = 17024)			2949713 (N^a^ = 78)	693976 (N^a^ = 234)	
Overall proportion ^d^	17201	1.0 (0.7, 1.2)	99.0 (98.8, 99.3)	<0.001	312	19.0 (13.1, 25.0)	81.0 (75.0, 86.9)	<0.001
**Gender**								
Female	8995	51.0 (50.2, 51.8)	27.7 (17.7,37.4)	<0.001	110	37.4 (30.9,43.9)	18.8 (8.0, 29.6)	0.014
Male	8206	49.0 (48.2, 49.7)	72.4 (62.6, 82.3)		202	62.6 (56.2, 69.3)	81.2 (70.4, 92.0)	
**Race**								
Non-Hispanic White	6307	61.7 (58.4, 64.9)	67.7 (58.8, 76.7)	0.03	205	82.7 (78.0, 87.4)	73.6 (61.6, 85.5)	<0.001
Hispanic	4559	17.5 (14.9, 20.0)	10.2 (5.6, 14.8)		47	8.1 (5.0, 11.6)	11.6 (3.9, 19.2)	
Non-Hispanic Black	3629	11.7 (10.1, 13.3)	16.3 (10.1, 22.6)		34	2.4 (1.0, 3.9)	13.1 (5.3, 20.9)	
Others	2706	9.2 (8.2, 10.2)	5.7 (1.4, 10.0)		26	6.7 (3.4, 10.1)	1.6 (0, 3.8)	
**Age group**								
20–39	8616	49.8 (48.4, 51.3)	13.4 (6.7, 20.1)	<0.001	124	45.1 (37.6, 52.5)	20.2 (8.4, 32.0)	<0.001
40–59	8585	50.2 (48.7, 51.6)	86.6 (80.0, 93.3)		188	55.0 (47.5, 62.4)	79.8 (68.1, 91.6)	
**Age (in years)** Mean (95% CI)	17201	49.1 (47.4, 50.8	39.5 (39.1, 39.8)	<0.001	312	48.8 (46.1, 51.5)	41.3 (39.6, 43.1)	<0.001
**Education**								
≤ High school grad	7323	36.0 (33.9, 38.1)	67.0 (58.1, 75.9)	<0.001	172	48.1 (40.3, 55.8)	74.0 (61.4, 84.5)	0.006
Some college degree	5506	32.7 (31.5, 34.0)	26.1 (18.2, 34.1)		117	41.9 (34.7, 49.1)	20.7 (9.2, 32.1)	
≥ College grad	4361	31.3 (29.0, 33.6)	6.9 (0.4, 13.4)		23	10.1 (5.2, 14.9)	5.4 (0, 12.7)	
Missing	11				0			
**Poverty Income Ratio**								
<1 (Poor)	3730	16.2 (14.8, 17.6)	33.6 (24.4, 42.9)	<0.001	123	26.3 (19.6, 33.1)	38.7 (23.6, 53.7)	0.29
1–1.99 (Near poor)	4032	20.0 (18.9, 21.2)	32.7 (21.8, 43.5)		84	25.4 (17.5, 33.2)	19.1 (8.8, 29.5)	
≥2 (Not poor)	7912	63.8 (61.7, 65.8)	33.7 (20.9, 46.5)		92	48.3 (39.4, 52.2)	42.2 (22.6, 61.8)	
Missing	1527				13			
**Health Insurance**								
No	4702	21.3 (19.8, 22.9)	38.3 (18.7, 47.9)	<0.001	118	30.8 (23.4, 38.1)	43.4 (28.2, 58.7)	0.118
Yes	12477	78.7 (77.1, 80.2)	61.7 (52.1, 71.3)		194	69.2 (61.9, 76.6)	56.6 (41.3, 71.8)	
Missing	22				9			
**Blood transfusion**								
No	15860	93.4 (92.8, 93.9)	83.2 (76.2, 90.2)	<0.001	272	92.1 (87.0, 97.3)	87.4 (78.8, 96.0)	0.337
Yes	1207	6.7 (6.1, 7.2)	16.8 (9.8, 23.9)		31	7.9 (2.7, 3.1)	12.6 (4.0, 21.2)	
Missing	134				9			
**Injection drug use**								
No	14894	97.9 (97.5, 98.3)	50.3 (38.3, 62.4)	<0.001				
Yes	312	2.1 (1.7, 2.5)	49.7 (37.6, 61.8)					
Missing	1995							
**Hepatitis B status**								
Never infected	11619	68.3 (67.1, 69.5)	61.8 (52.0, 71.6)	<0.001	195	68.0 (60.9, 75.1)	59.0 (44.2, 73.8)	0.027
Current/Past infection	1065	3.8 (3.3, 4.2)	27.1 (19.4,34.9)		61	11.9 (6.9, 16.9)	29.1 (17.1, 41.0)	
Vaccine-Induced Immunity	4517	28.0 (26.9, 29.1)	11.1 (4.6, 17.6)		56	20.1 (15.0, 25.3)	1.0 (22.9)	
**Number of Sexual Partner last 12 months**								
0	1230	17.1 (15.9, 18.4)	37.7 (19.6, 55.8)	0.005	24	13.4 (4.4, 22.5)	31.1 (10.9, 51.3)	0.243
1	4495	69.0 (67.6, 70.4)	48.7 (32.4, 65.0)		58	56.0 (41.2, 70.7)	45.9 (25.9, 65.9)	
≥2	1018	13.9 (12.8, 15.0)	13.6 (4.2, 23.0)		33	30.6 (17.5, 43.7)	23.1 (6.5, 39.6)	
Missing	10458				197			
**HIV infection**								
No	16888	99.6 (99.4, 99.7)	97.5 (94.5, 100.0)	0.001				
Yes	89	0.4 (0.3, 0.6)	2.5 (0.0, 5.5)					
Missing	224							
**Diabetes**								
No	15614	93.8 (93.2, 94.3)	90.4 (84.9, 95.9)	0.15	280	93.8 (89.6, 98.0)	85.4 (74.8, 96.0)	0.106
Yes	1277	6.2 (5.7, 6.8)	9.6 (4.1, 15.2)		27	6.2 (2.0, 10.4)	14.6 (4.0, 25.2)	
Missing	310				5			

General population includes both PWID & non-PWID.

^a^ N: Unweighted sample size (i.e., analytical sample size).

^b^ Weighted prevalence estimates in percent (Wt%) and 95% confidence intervals (CI) were obtained by applying NHANES sampling.

weights. And, projected population is the weighted N.

^c^ P-value from Rao-Scott Chi-square tests for categorical predictors or weighted linear regression for continuous predictors.

^d^ P-value for comparing total CHI positive vs negative proportions was obtained from Z-test.

Weighted percentage of CHI by HIV infection status for PWID was not calculated due to limited sample size.

### Prevalence of CHI in the general population and PWID

The overall prevalence of CHI was 1.0% in the general population including both PWID and non-PWID, noticeably high at 19% among only PWID (Table 2). In the general population, the prevalence was 0.5% among females and 1.4% among males, while among PWID, the prevalence of CHI was 10.6% among females and 23.4% among males. When considering race, the prevalence of CHI was higher among Non-Hispanic Black individuals, with rates of 1.3% in the general population and 55.8% among PWID. Conversely, the prevalence was lowest among Hispanics in the general population (0.6%) and among other groups in the PWID population (5.8%). Among both the general population and PWID, individuals in the age group of 40–59 years had the highest prevalence of CHI, with rates of 1.7% and 25.5%, respectively. Among the HIV infected general population, the prevalence of CHI was 5.5%. Participants with current or past HBV infection had the highest CHI prevalence in both the general population and only PWID (6.6% and 36.5%, respectively). When PWID were defined by IDU within the last 12 months, the prevalence of CHI was 24.7%, approximately 5% higher than the 19% prevalence observed when PWID were defined by ever use, as shown in S1 Table. However, the sample size of PWID participants was noticeably smaller (N = 67) compared to those defined by ever use (N = 302).

**Table 2:** Prevalence of CHI by characteristics in general population and PWID.

		General Population		People Who Inject Drugs
Characteristics	N^a^	Weighted % (95% CI)^a^	N^a^	Weighted % (95% CI)^a^
N^b^		N = 177		N = 78
Overall proportion	17201	1.0 (.7, 1.2)	312	19.0 (13.1, 25.0)
**Gender**				
Female	8995	0.5 (0.3, 0.7)	110	10.6 (5.4, 15.8)
Male	8206	1.4 (1.0, 1.8)	202	23.4 (14.8, 31.9)
**Race**				
Non-Hispanic White	6307	1.1 (0.7, 1.4)	205	17.3 (10.3, 24.4)
Hispanic	4559	0.6 (0.4, 0.8)	47	25.1 (9.8, 40.3)
Non-Hispanic Black	3629	1.3 (0.9, 1.8)	34	55.8 (37.1, 74.5)
Others	2706	0.6 (0.1, 1.1)	26	5.8 (0, 13.4)
**Age group**				
20–39	8616	0.3 (0.1, 0.4)	124	9.5 (4.0, 15.1)
40–59	8585	1.7 (1.3, 2.1)	188	25.5 (17.0, 33.9)
**Education**				
≤ High school grad	7323	1.8 (1.4, 2.2)	172	26.6 (17.8, 35.4)
Some college degree	5506	0.8 (0.5, 1.0)	117	10.4 (4.8, 16.0)
≥ College grad	4361	0.2 (0.0, 0.4)	23	11.2 (0.0, 25.1)
**Poverty Income Ratio**				
<1 (Poor)	3730	2.0 (1.5, 2.5)	123	25.7 (17.7, 33.7)
1–1.99 (Near poor)	4032	1.6 (1.0, 2.2)	84	15.1 (7.4, 22.8)
≥2 (Not poor)	7912	0.5 (0.2, 0.8)	92	17.1 (6.1, 28.1)
**Health Insurance**				
No	4702	1.7 (1.2, 2.2)	118	24.9 (16.8, 33.1)
Yes	12477	0.8 (0.5, 1.0)	194	16.1 (8.5, 23.7)
**Blood transfusion**				
No	15860	0.9 (0.6, 1.1)	272	18.5 (12.2, 24.8)
Yes	1207	2.4 (1.3, 3.4)	31	27.6 (7.4, 47.9)
**Injection drug use**				
No	14894	0.5 (0.4, 0.7)		
Yes	312	19.1 (13.1, 25)		
**Hepatitis B status**				
Never infected	11619	0.9 (0.6, 1.1)	195	16.9 (9.8, 24.1)
Current/Past infection	1065	6.6 (4.2, 8.9)	61	36.5 (22.1, 50.9)
Vaccine-Induced Immunity	4517	0.4 (0.2, 0.6)	56	12.3 (0.6, 23.9)
**Number of Sexual Partner last 12 months**				
0	1230	1.8 (0.6, 3.0)	24	31.1 (7.3, 54.9)
1	4495	0.6 (0.3, 0.8)	58	13.8 (4.2, 23.4)
≥2	1018	0.8 (0.2, 1.4)	33	12.8 (1.7, 24.0)
**HIV infection**				
No	16888	0.9 (0.7, 1.2)		
Yes	89	5.5 (0.0, 11.8)		
**Diabetes**				
No	15614	0.9 (0.7, 1.2)	280	17.7 (11.3, 24.1)

General population includes both PWID & non-PWID.

^a^ Weighted prevalence estimates in percent and 95% confidence intervals (CI) were obtained by applying NHANES sampling weights.

^b^ N: Number of CHI-positive participants in general population and PWID.

### Associated factors of CHI in the general population

The current study employed univariate and multivariable logistic regression models to investigate the associated risk factors of CHI. Seven of the eight risk factors, including gender, age, race, education, poverty income ratio, blood transfusion, and IDU, demonstrated significance (p<0.05) in both univariate and multivariable logistic regressions (Table 3). Hence, these factors independently (without the influence of adjusting factors) contributed to the likelihood of CHI in the general population. The adjusted odds ratio (AOR) from the multivariable model was interpreted. Males exhibited approximately twice higher odds of having CHI than females. Adults aged 40–59 years were 6.4 (95% CI = 3.2–12.6) times more likely to have CHI than those aged 20–39 years. Among different racial groups, the adjusted odds of having CHI were 50% (95% CI = 0.3–0.9) lower among Hispanics and 1.6 (95% CI = 0.9–2.8) times higher among non-Hispanic blacks compared to non-Hispanic whites. Furthermore, individuals with a high school education or below had approximately twice higher odds of having CHI than those with an education level above college (95% CI = 1.3–4.4). In terms of the poverty income ratio, the poor and near-poor categories had 3.2 times higher odds of having CHI (95% CI = 1.7–6.1 and 1.5–7.0, respectively) compared to the not poor participants. The odds of having CHI were 2.6 (95% CI = 1.4–4.9) times higher among individuals with a history of blood transfusion compared to those who did not have a history of blood transfusion.

**Table 3:** Factors associated with current HCV infection in general population and PWID.

	General population				People who inject drugs			
	COR (95% CI)	P-value ^a^	AOR (95% CI)	P-value^b^	COR (95% CI)	P-value ^a^	AOR (95% CI)	P-value^b^
**Gender**								
Female (ref)								
Male	2.7 (1.7, 4.5)	<0.001	2.2 (1.2, 3.9)	0.01	2.6 (1.2, 5.5)	0.017	2.6 (1.1, 5.9)	0.03
**Age group**								
20–39 (ref)								
40–59	6.4 (3.6, 11.4)	<0.001	6.4 (3.2, 12.6)	<0.001	3.2 (1.5, 6.9)	0.004	2.9 (1.2, 7.3)	0.029
**Race**								
Non-Hispanic white (ref)								
Hispanic	0.5 (0.3, 0.9)	0.014	0.5 (0.3, 0.9)	0.02	1.6 (0.7, 3.9)	0.304	1.4 (0.6, 3.6)	0.443
Non-Hispanic black	1.3 (0.8, 2.1)	0.342	1.6 (0.9, 2.8)	0.093	6.0 (2.5, 14.6)	<0.001	4.6 (1.5, 13.6)	0.01
Others	0.6 (0.3, 1.3)	0.173	1.1 (0.4, 2.8)	0.905	0.3 (0.1, 1.4)	0.122	0.4 (0.1, 1.4)	0.16
**Education**								
≤ High School Graduate	2.3 (1.6, 3.5)	<0.001	2.4 (1.3, 4.4)	0.006	3.1 (1.5, 6.5)	0.004	3.5 (1.4, 9.2)	0.014
≥ College graduate	0.3 (0.1, 0.8)	0.021	0.6 (0.2, 1.9)	0.354	1.1 (0.2, 4.9)	0.912	1.3 (0.3, 5.2)	0.766
Above college degree (ref)								
**Poverty Income Ratio**								
<1 (poor)	3.9 (2.2, 6.9)	<0.001	3.2 (1.7, 6.1)	0.001	1.7 (0.7, 4.0)	0241	1.3 (0.5, 3.3)	0.588
1–1.99 (near poor)	3.1 (1.6, 6.0)	0.001	3.2 (1.5, 7.0)	0.005	0.9 (0.3, 2.3)	0.771	0.9 (0.3, 2.9)	0.894
≥2 (not poor) [ref]								
**Blood transfusion**								
No (ref)					-	-	-	-
Yes	2.4 (1.6, 3.6)	<0.001	2.6 (1.4, 4.9)	0.004	1.1 (0.5, 2.4)	0.838	2.2 (0.7, 6.8)	0.195
**Injection drug use**								
No (ref)								
Yes	47.9 (32.2, 71.3)	<0.001	32.6 (17.7, 60.3)	<0.001	-	-	-	-
**HIV infection**								
No (ref)								
Yes	6.1 (1.8, 20.2)	0.004	0.8 (0.1, 4.5)	0.794	-	-	-	-

COR: Crude Odds Ratio from univariate logistic regression; AOR: Adjusted Odds Ratio from multivariable logistic regression. General population includes both PWID & non-PWID.

^a^ P value from univariate logistic regression.

^b^ P value from multivariable logistic regression.

Variable selection criteria for multivariable models: either significant in univariate analysis or deemed clinically important for active HCV infection. HIV status was not included in the separate analysis on PWID due to limited sample size.

The findings of this study highlight the noteworthy fact that individuals who engaged in IDU had a significantly higher likelihood of having CHI, with odds that are 32.6 (95% CI = 17.7–60.3) times greater than those who did not partake in drug injection. Similar findings were observed when the PWID were defined by the IDU within last 12 months (S2 Table).

### Associated factors for CHI among PWID

Univariate and multivariable logistic regression was used to analyze the factors influencing HCV status among PWID from 2009 to 2018 (Table 3). Four of the six predictors (gender, age group, race, and education) were significant in both univariate and multivariable logistic regressions. The odds of CHI were 2.6 (95% CI = 1.1–5.9) times higher in males than females. In addition, adults aged 40–59 years were 2.9 (95% CI = 1.2–7.3) times more likely to have a CHI than adults aged 20–39. With race, the adjusted odds of having CHI were 40% (95% CI = 0.6–3.6) higher among Hispanics, 4.6 (95% CI = 1.5–13.6) times higher among non-Hispanic blacks, and 60% (95% CI = 0.1–1.4) lower among others compared to non-Hispanic whites. In terms of education, individuals with a high school education or below had 3.5 (95% CI = 1.4–9.5) times higher odds of CHI compared to those with an education above the college level. Conversely, those with a college or higher degree had 1.3 (95% CI = 0.3–5.2) times higher odds.

## Discussion

In this study, we conducted an assessment of the prevalence of CHI and associated risk factors among both general population and PWID in the United States, using the most recent nationally representative data available. Our findings revealed that the prevalence of CHI is notably higher among PWID in general population, particularly among males, adult aged 40–59 years, Non-Hispanic Black individuals, and those with a high school education or less. Additionally, individuals with a history of blood transfusion and those living below the poverty line were at a heightened risk of CHI. These trends align with prior studies conducted in various regions. For instance, research in rural northeastern US [24] and New Mexico [25] found that individuals who inject drugs, or those with a positive history of drug use, particularly opioids, were at an increased risk of HCV infection. A study in Hawaii highlighted PWID, individuals with a history of blood transfusion, and males [26] as an independent risk factor for HCV infection, which corroborates our findings. Another study, which focused on populations in eight US cities in 2015, noted a higher prevalence of HCV antibody positivity among PWID aged over 35 years [27]. In terms of racial disparities, previous research has indicated that African Americans not only exhibit lower responsiveness to anti-HCV therapy but also are less likely to naturally clear HCV, potentially contributing to the higher prevalence of HCV [28] as well as increased transmission rates in this demographic group. This finding also aligns with our study’s results, which indicate a higher prevalence and increased odds of CHI in this group. Furthermore, studies [29,30] both within and outside the US have identified PWID and prison tattooing as risk factors for HCV infection.

Importantly, these findings emphasize the necessity of targeted interventions, particularly among vulnerable group such as PWID, to prevent HCV infection [29]. Harm reduction programs should extend beyond routine HCV screening, encompass awareness campaigns promoting safe injection practices, educational programs, and psychological support for individuals dealing with the root causes of drug injection. These measures not only reduce the risk of hepatitis C infection, but also contribute to preventing related complications, such as bile duct cancer or liver cirrhosis [30]. Additionally, our results indicate that individuals with less than a high school education are at a higher risk, and this aligns with a previous study that found they were less likely to get tested for HCV infection [31]. Therefore, it’s essential to increase HCV testing among this demographic. Community-based health education programs should be implemented to raise awareness and knowledge about HCV infection and its risks.

In our study, we identified independent risk factors associated with CHI among PWID, including male gender, Non-Hispanic Black ethnicity, age between 40–49 years, and having a high school education or less. A study conducted in Miami [32] found similar trends, with lower school education levels, and increasing age significantly correlating with HCV infection, though the study did not find significant differences by race and gender. However, it’s important to note that these discrepancies might be attributed to the limitations of a single syringe services program survey conducted in one city, which cannot be generalized to the broader PWID population in the US. Additionally, a cross-sectional study (2005–2012) [33] and qualitative study [34] done in New York City among young PWID highlighted additional risk factors for HCV infections. These included sharing syringes, injecting in outdoor or public spaces, and being arrested for carrying syringes. A systematic review study [35] revealed that factors such as being female, age over 30 years, and having previous treatment attempts were more likely to have HCV testing. Conversely, not practicing safe sex and residing in rural areas were associated with a lower likelihood of HCV testing [36] among PWID. Prior research often focused on single urban areas or conducted literature reviews, underscoring the need for interventions programs aimed at changing behavioral practices to promote the use of safe syringes and ensure a consistent supply of sterile injection equipment to prevent HCV transmission. Encouraging PWID especially men who are more at risk, to seek HCV testing is vital. However, to our knowledge, there have been no recent studies examining nationally representative data on HCV infection. Our study, addressed this gap by examining a larger at risk population aged 20–59 years, aiming to identify more targeted interventions for both general population and specifically PWID. Based on our findings, we can strategize different interventions programs to prevent HCV infection. For examples, policies aimed at HCV screening programs, particularly for Black individuals, should be improved, with a focus on males aged 40–49 years old. Efforts to pursue higher educational degree among all youths are essential. Moreover, health education programs on HCV should be conducted in the community, not only for PWID at risk but also for drug treatment staff to prevent accidents that can occur during medical procedures. These findings underscore the critical need for enhanced public health initiatives and targeted interventions to mitigate the impact of HCV within high-risk populations.

Our findings support the Centers for Disease Control and Prevention recommendation for universal HCV screening among adults aged 18 and older [37], with a high prevalence among PWID, particularly males, adults aged 40–59, Non-Hispanic Black individuals, and those with less than high school education. Moreover, our findings can help target screening efforts more effectively. Our results align with the recommendation by the American Association for the Study of Liver Diseases (AASLD) and Infectious Diseases Society of America (IDSA) [38] regarding the need for HCV screening and treatment of PWID. Strategies remain necessary that are focused on this high-risk population, with the removal of obstacles to care-especially through multidisciplinary settings, as recently recommended by AASLD/IDSA. Our findings have significant implications for public health in relation to the identification of vulnerable subgroups, harm reduction strategies, including needle exchange programs and opioid agonist therapy, and policy decisions about resource allocation. Further research into effective strategy and policy formulation in line with evidence-based interventions is required for new screening methods and treatment adherence strategies for PWID. Our results thus contribute to a growing body of literature on the HCV prevalence among PWID, emphasizing targeted and specific interventions at key points while pointing forward to other avenues of research and policy development for HCV elimination.

Additionally, we acknowledged that PWID group faces an elevated risk of contracting other sexually transmitted diseases such as HIV, which accounts for at least 10% of new HIV cases [39]. This highlights the potential for coinfection of HIV or other blood borne infections alongside HCV [40]. Furthermore, PWID are often exposed to multiple adverse risk environments that exacerbate health risks [41]. Despite substantial evidence indicating that behavioral interventions can mitigate the risk of new infections among PWID, there exists a significant research gap, largely driven by the socioeconomic and racial disparities present in society. Addressing these disparities and reshaping the social structure are essential steps toward achieving health equity and reducing the prevalence of PWID in society. Future studies on behavioral health interventions among PWID are, therefore, of utmost importance.

The strength of this study is that we used a large and nationally representative sample. This means that our results can be generalized to the respective U.S. populations aged 20–59 years. However, we acknowledge some limitations. The cross-sectional nature of study prevents us from establishing causality for the observed associations. Additionally, the self-reporting of drug injection practices may introduce the possibility of misreporting by individuals who inject drugs. We also lack information regarding non-licensed tattoo providers, and the HCV status of their partners, both of which are risk factors for HCV infection [42]. Further research is warranted to elucidate these associations, understand the underlying mechanisms, and identify implications for the development of targeted interventions for high-risk populations.

## Conclusion

The prevalence of CHI is higher among individuals who engaged in IDU in the general population and especially among PWID who were males, adults aged 40–59 years old, Non-Hispanic Black, and had educational attainment at or below a high school diploma were more vulnerable to have CHI infection. Thus, targeted intervention such as screening and awareness program for vulnerable PWID is recommended to reduce the burden of HCV infection and mitigate related health complications. Further research is warranted regarding the development of effective strategies and policies to prevent the HCV infection.

## Supporting information

S1 Checklist STROBE statement—checklist of items that should be included in reports of observational studies. (DOCX)

S1 Table Prevalence of CHI by IDU categories, with PWID defined by last 12 months injection drug use. (DOCX)

S2 Table Factors associated with current HCV infection in general population, with PWID defined by injection drug use in the past 12 months. (DOCX)
