# Supplementary material for: Prevalence and associated risk factors of current hepatitis C infection among U.S. general population and injection drug users aged 20–59 years: NHANES 2009–2018
Source: PLoS One. 2024 Aug 26;19(8):e0309345. doi: 10.1371/journal.pone.0309345 (PMC11346729; doi:10.1371/journal.pone.0309345)
Supplement: S1 Checklist — (DOCX) [file pone.0309345.s001.docx]

STROBE Statement—checklist of items that should be included in reports of observational studies

|  | Item No. | Recommendation | Page  No. | Relevant text from manuscript |
| --- | --- | --- | --- | --- |
| **Title and abstract** | 1 | (*a*) Indicate the study’s design with a commonly used term in the title or the abstract | 1 | This study utilized cross-sectional data from the 2001-2018 National Health and Nutrition Examination Survey and conducted separate analyses for the U.S. |
|  |  | (*b*) Provide in the abstract an informative and balanced summary of what was done and what was found | 1-2 | The prevalence of CHI among general population and PWID were 1.2% and 22.7%, respectively. Compared to non-PWID, the odds of CHI were significantly higher among PWID (OR=33.8, 95% CI=21.4-53.5) in general population… |
| Introduction | | | |  |
| Background/rationale | 2 | Explain the scientific background and rationale for the investigation being reported | 3 | Infection with the hepatitis C virus (HCV) is a major concern in the U.S. and globally which contributes to significant mortality and morbidity… |
| Objectives | 3 | State specific objectives, including any prespecified hypotheses | 4 | This study aims to determine the prevalence of CHI status among the general population and PWID in the U.S..… |
| Methods | | | |  |
| Study design | 4 | Present key elements of study design early in the paper | 4-5 | Data were collected from National Health and Nutrition Examination Survey (NHANES) 2001-2018… |
| Setting | 5 | Describe the setting, locations, and relevant dates, including periods of recruitment, exposure, follow-up, and data collection | 4-5 | Data were collected from National Health and Nutrition Examination Survey (NHANES) 2001-2018… |
| Participants | 6 | (*a*) *Cohort study*—Give the eligibility criteria, and the sources and methods of selection of participants. Describe methods of follow-up  *Case-control study*—Give the eligibility criteria, and the sources and methods of case ascertainment and control selection. Give the rationale for the choice of cases and controls  *Cross-sectional study*—Give the eligibility criteria, and the sources and methods of selection of participants | 5 | The general population included participants with or without a history of IDU, which allowed the evaluation risk attributes in PWID relative to non-PWID... |
|  |  | (*b*) *Cohort study*—For matched studies, give matching criteria and number of exposed and unexposed  *Case-control study*—For matched studies, give matching criteria and the number of controls per case | NA | NA |
| Variables | 7 | Clearly define all outcomes, exposures, predictors, potential confounders, and effect modifiers. Give diagnostic criteria, if applicable | 5 | The response variable was the current HCV infection status (CHI): positive or negative. Participants diagnosed as positive in HCV RNA-test were identified as CHI “positive”. Predictors included: age (20-39, 40-59)… |
| Data sources/ measurement | 8* | For each variable of interest, give sources of data and details of methods of assessment (measurement). Describe comparability of assessment methods if there is more than one group | *5* | The response variable was the current HCV infection status (CHI): positive or negative. Participants diagnosed as positive in HCV RNA-test were identified as CHI “positive”. Predictors included: age (20-39, 40-59)… |
| Bias | 9 | Describe any efforts to address potential sources of bias | NA | NA |
| Study size | 10 | Explain how the study size was arrived at | 4-5 | The analytical sample size was 39689 for the general population where 759 participants were PWID (Figure 1).… |

Continued on next page

| Quantitative variables | 11 | Explain how quantitative variables were handled in the analyses. If applicable, describe which groupings were chosen and why | 5-6 | Predictors included: age (20-39, 40-59), gender (male, female), race (Hispanic, Non-Hispanic white, Non-Hispanic black, and others), education (high school graduate or less, some college degree, some college or above), poverty income ratio (<1: poor, 1-1.99: near poor, ≥2: not poor),… |
| --- | --- | --- | --- | --- |
| Statistical methods | 12 | (*a*) Describe all statistical methods, including those used to control for confounding | 6 | All analyses in this study were adjusted by appropriate NHANES sampling weights (“WTMEC2yr”) to ensure nationally representative estimates … |
|  |  | (*b*) Describe any methods used to examine subgroups and interactions | 6 | The above analyses were conducted for both the general population and PWID … |
|  |  | (*c*) Explain how missing data were addressed | NA | NA |
|  |  | (*d*) *Cohort study*—If applicable, explain how loss to follow-up was addressed  *Case-control study*—If applicable, explain how matching of cases and controls was addressed  *Cross-sectional study*—If applicable, describe analytical methods taking account of sampling strategy | 5 | All analyses in this study were adjusted by appropriate NHANES sampling weights (“WTMEC2yr”) to ensure nationally representative estimates … |
|  |  | (*e*) Describe any sensitivity analyses | NA |  |
| Results | | | | |
| Participants | 13* | (a) Report numbers of individuals at each stage of study—eg numbers potentially eligible, examined for eligibility, confirmed eligible, included in the study, completing follow-up, and analysed | 6 | Fig 1  Table 1 displays the background characteristics of the study participants … |
|  |  | (b) Give reasons for non-participation at each stage | NA | Fig 1 |
|  |  | (c) Consider use of a flow diagram | 7 | Fig 1 |
| Descriptive data | 14* | (a) Give characteristics of study participants (eg demographic, clinical, social) and information on exposures and potential confounders | 7-8 | Table 1 shows the number of participants … |
|  |  | (b) Indicate number of participants with missing data for each variable of interest | NA | NA |
|  |  | (c) *Cohort study*—Summarise follow-up time (eg, average and total amount) |  |  |
| Outcome data | 15* | *Cohort study*—Report numbers of outcome events or summary measures over time |  |  |
|  |  | *Case-control study—*Report numbers in each exposure category, or summary measures of exposure |  |  |
|  |  | *Cross-sectional study—*Report numbers of outcome events or summary measures | *6* | *Among 39,689 individuals from the general population, 504 tested positive for CIH, while 39,185 tested negatives* |
| Main results | 16 | (*a*) Give unadjusted estimates and, if applicable, confounder-adjusted estimates and their precision (eg, 95% confidence interval). Make clear which confounders were adjusted for and why they were included | 7-10 | Table 2 and 3…. |
|  |  | (*b*) Report category boundaries when continuous variables were categorized | 7-10 | Table 1, 2 and 3 |
|  |  | (*c*) If relevant, consider translating estimates of relative risk into absolute risk for a meaningful time period |  |  |

Continued on next page

| Other analyses | 17 | Report other analyses done—eg analyses of subgroups and interactions, and sensitivity analyses |  |  |
| --- | --- | --- | --- | --- |
| Discussion | | | | |
| Key results | 18 | Summarise key results with reference to study objectives | 11-12 | Our findings revealed that the prevalence of CHI is notably higher among PWID in general population, particularly among males, adult aged 40-59 years, Non-Hispanic Black individuals, and those with a high school education or less…. |
| Limitations | 19 | Discuss limitations of the study, taking into account sources of potential bias or imprecision. Discuss both direction and magnitude of any potential bias | 12-14 | The strength of this study is that we used a large and nationally representative sample. This means that our results can be generalized to the respective U.S. populations aged 20-59 years. However, we acknowledge some limitations. The cross-sectional nature of study prevents us from establishing causality for the observed associations. …. |
| Interpretation | 20 | Give a cautious overall interpretation of results considering objectives, limitations, multiplicity of analyses, results from similar studies, and other relevant evidence | 11-12 | Importantly, these findings emphasize the necessity of targeted interventions, particularly among vulnerable group such as PWID, to prevent HCV infection …. |
| Generalisability | 21 | Discuss the generalisability (external validity) of the study results | 11-12 | we identified independent risk factors associated with CHI among PWID, including male gender, Non-Hispanic Black ethnicity, age between 40-49 years, and having a high school education or less…. |
| Other information | |  | | |
| Funding | 22 | Give the source of funding and the role of the funders for the present study and, if applicable, for the original study on which the present article is based | 13 | The author(s) received no specific funding for this work. |
|  |  |  |  |  |

*Give information separately for cases and controls in case-control studies and, if applicable, for exposed and unexposed groups in cohort and cross-sectional studies.

**Note:** An Explanation and Elaboration article discusses each checklist item and gives methodological background and published examples of transparent reporting. The STROBE checklist is best used in conjunction with this article (freely available on the Web sites of PLoS Medicine at http://www.plosmedicine.org/, Annals of Internal Medicine at http://www.annals.org/, and Epidemiology at http://www.epidem.com/). Information on the STROBE Initiative is available at www.strobe-statement.org.
