# Supplementary material for: Prevalence and associated risk factors of current hepatitis C infection among U.S. general population and injection drug users aged 20–59 years: NHANES 2009–2018
Source: PLoS One. 2024 Aug 26;19(8):e0309345. doi: 10.1371/journal.pone.0309345 (PMC11346729; doi:10.1371/journal.pone.0309345)
Supplement: S1 Table — (DOCX) [file pone.0309345.s002.docx]

S1 Table: Prevalence of CHI by IDU categories, with PWID defined by last 12 months injection drug use.

|  |  | **General Population** |
| --- | --- | --- |
| **Characteristics** | **N^a^** | **Weighted % (95% CI)^a^** |
| N^b^ |  | N = 177 |
| Overall proportion | 17201 | 1.0 (.7, 1.2) |
| **Injection drug use**  **(last 12 months)** |  |  |
| No | 15138 | 0.9 (0.6, 1.1) |
| Yes | 67 | 24.7 (12.7, 36.7) |
| General population includes both PWID & non-PWID.  ^a^ Weighted prevalence estimates in percent and 95% confidence intervals (CI) were  obtained by applying NHANES sampling weights.  ^b^ N: Number of CHI-positive participants. | | |
