# Supplementary material for: Prevalence and associated risk factors of current hepatitis C infection among U.S. general population and injection drug users aged 20–59 years: NHANES 2009–2018
Source: PLoS One. 2024 Aug 26;19(8):e0309345. doi: 10.1371/journal.pone.0309345 (PMC11346729; doi:10.1371/journal.pone.0309345)
Supplement: S2 Table — (DOCX) [file pone.0309345.s003.docx]

S2 Table: Factors associated with current HCV infection in general population, with PWID defined by injection drug use in the past 12 months.

|  | **General population** | | | |
| --- | --- | --- | --- | --- |
|  | **COR (95% CI)** | **P-value ^a^** | **AOR (95% CI)** | **P-value ^b^** |
| **Gender** |  |  |  |  |
| Female (ref) |  |  |  |  |
| Male | 2.7 (1.7, 4.5) | <0.001 | 2.7 (1.5, 4.8) | <0.001 |
| **Age group** |  |  |  |  |
| 20-39 (ref) |  |  |  |  |
| 40-59 | 6.4 (3.6, 11.4) | <0.001 | 8.7 (4.4, 17.1) | 0.002 |
| **Race** |  |  |  |  |
| Non-Hispanic white (ref) |  |  |  |  |
| Hispanic | 0.5 (0.3, 0.9) | 0.014 | 0.3 (0.2, 0.6) | <0.001 |
| Non-Hispanic black | 1.3 (0.8, 2.1) | 0.342 | 1.1 (0.7, 1.9) | 0.66 |
| Others | 0.6 (0.3, 1.3) | 0.173 | 0.7 (0.3, 1.9) | 0.517 |
| **Education** |  |  |  |  |
| $\leq$High School Graduate | 2.3 (1.6, 3.5) | <0.001 | 2.7 (1.5, 4.6) | 0.001 |
| $\geq$ College graduate | 0.3 (0.1, 0.8) | 0.021 | 0.5 (0.1, 1.6) | 0.227 |
| Above college degree (ref) |  |  |  |  |
| **Poverty Income Ratio** |  |  |  |  |
| <1 (poor) | 3.9 (2.2, 6.9) | <0.001 | 4.0 (2.1, 7.6) | <0.001 |
| 1-1.99 (near poor) | 3.1 (1.6, 6.0) | 0.001 | 2.8 (1.3, 6.0) | 0.008 |
| $\geq$2 (not poor) [ref] |  |  |  |  |
| **Blood transfusion** |  |  |  |  |
| No (ref) |  |  |  |  |
| Yes | 2.8 (1.7, 4.7) | <0.001 | 2.4 (1.4, 4.1) | 0.003 |
| **Injection drug use (Last 12 Months)** |  |  |  |  |
| No (ref) |  |  |  |  |
| Yes | 37.7 (18.2, 78.3) | <0.001 | 38.1 (15.5, 93.7) | <0.001 |
| **HIV** |  |  |  |  |
| No |  |  |  |  |
| Yes (ref) | 6.1 (1.8, 20.2) | 0.004 | 0.4 (0.1, 2.5) | 0.328 |
| COR: Crude Odds Ratio from univariate logistic regression; AOR: Adjusted Odds Ratio from multivariable logistic regression. General population includes both PWID & non-PWID.  ^a^ P value from univariate logistic regression  ^b^ P value from multivariable logistic regression  Variable selection criteria for multivariable models: either significant in univariate analysis or deemed clinically important for active HCV infection. A separate analysis on PWID was not conducted due to limited sample size in several subgroups within PWID. | | | | |
